# Risk network approaches to locating undiagnosed HIV cases in Odessa, Ukraine

**DOI:** 10.1002/jia2.25040

**Published:** 2018-01-22

**Authors:** Pavlo Smyrnov, Leslie D Williams, Ania Korobchuk, Yana Sazonova, Georgios K Nikolopoulos, Britt Skaathun, Ethan Morgan, John Schneider, Tetyana I Vasylyeva, Samuel R Friedman

**Affiliations:** ^1^ Alliance for Public Health Kyiv Ukraine; ^2^ National Development and Research Institutes New York NY USA; ^3^ Alliance for Public Health Odessa Ukraine; ^4^ Medical School University of Cyprus Nicosia Cyprus; ^5^ University of Chicago Chicago IL USA; ^6^ Division of Global Public Health University of California San Diego CA USA; ^7^ Department of Medicine and Center for HIV Elimination University of Chicago Chicago IL USA; ^8^ Department of Zoology University of Oxford Oxford United Kingdom; ^9^ Center for Drug Use and HIV Research New York NY USA

**Keywords:** HIV prevention, early infection, risk networks, social network, HIV diagnosis, treatment as prevention

## Abstract

**Introduction:**

Providing HIV healthcare and Treatment as Prevention both depend on diagnosing HIV cases, preferably soon after initial infection. We hypothesized that tracing risk networks recruits higher proportions of undiagnosed positives than outreach‐based testing or respondent‐driven sampling (RDS) in Odessa, Ukraine.

**Methods:**

The Transmission Reduction Intervention Project (TRIP) used risk network tracing to recruit sexual and injection networks of recently‐infected and longer‐term infected (LTs) seeds (2013 to 2016). Integrated Biobehavioural Surveillance (IBBS) (2013) used RDS to recruit people who inject drugs (PWID). Outreach Testing tested PWID for HIV at community outreach sites (2013 to 2016). Proportions of undiagnosed positives among those tested were compared TRIP versus IBBS; TRIP versus Outreach Testing and between TRIP arms. Costs were compared across the projects.

**Results:**

TRIP tested 1252 people (21% women) in seeds’ risk networks; IBBS tested 400 (18% women); Outreach Testing 13,936 (31% women). TRIP networks included a higher proportion of undiagnosed positives (14.6%) than IBBS (5.0%) or Outreach Testing (2.4%); odds ratio (OR) 3.25 (95% CI 2.07, 5.12) versus IBBS and 7.03 (CI 5.95, 8.31) versus Outreach Testing respectively. Findings remained significant in analyses stratified by sex and when PWID in TRIP networks were compared with Outreach Testing and IBBS. Within TRIP, recently‐infected participants’ networks contained higher proportions of undiagnosed positives (16.3%) than LTs’ networks (12.2%); OR 1.41 (CI 1.01, 1.95). TRIP located undiagnosed positives less expensively than did RDS or Outreach Testing.

**Conclusions:**

TRIP's recruiting techniques, including prioritizing networks of the recently infected, find undiagnosed HIV‐positive people efficiently. They should be integrated with standard practice to improve case‐finding. Research should test these techniques in other socio‐epidemiologic contexts.

**Clinical trial registry:**

Registered ClinicalTrials.gov: NCT01827228.

## 1 Introduction

Current global strategies to care for the infected and to limit HIV transmission depend to a large degree on locating and intervening with HIV‐infected persons. This has been concretized in the 90‐90‐90 strategy of having 90% of the infected know they are infected; 90% of those who know their status receiving treatment; and 90% of those in treatment having undetectable viral loads 1. Locating and diagnosing people who are HIV+ but undiagnosed is a crucial first part of the 90‐90‐90 strategy and central both for caring for the infected and for preventing further HIV transmission by them**.** Large‐scale testing and various forms of outreach to key populations are common strategies for locating undiagnosed positives.

In Ukraine and many other countries, both general and key population HIV testing projects spend considerable resources and test a very large number of HIV negatives for each undiagnosed positive they find. Social and risk network case‐finding has been found to help locate recently‐infected people 2, 3. We thus hypothesize that they should be able to locate more undiagnosed positives per test (and perhaps per staff member) than current testing approaches 2, 3, 4. This is because HIV is transmitted through risk (sexual and injection) networks, and those who are unaware that they are infected may be more likely to engage in condomless sex 5 or sharing of potentially‐infected injection paraphernalia. In addition, social norms and rumours about the advantages and disadvantages of HIV testing and HIV therapy are likely to spread and to be sustained in social networks (which often overlap with risk networks 6, 7, 8, 9, 10, 11).

The Transmission Reduction Intervention Project (TRIP) conducted network‐based recruiting, counseling and testing in Odessa, Ukraine, from November 2013 to March 2016. TRIP focused on locating recently‐infected people in order to prevent transmissions by them and their network members. Recently‐infected participants are an important target group for intervention because transmission is particularly likely during the period of early infection 12, 13, 14, 15, 16 due to high viral loads 1, 2, 3, 4, 5, 6, 7, 8, 9, 10, 11, 12, 13, 14, 15, 16, 17, 18, 19, lack of immune response, and perhaps‐temporary elevated rates of risky behaviours 12, 20. The full logic behind focusing on recently‐infected participants has been previously described 4, 15. Evidence from Athens (Greece) TRIP provides proof of concept that network recruiting can be effective in finding recently‐infected participants since they were more likely to be recruited in the risk networks of recently‐infected participants than in TRIP's comparison‐group networks of the longer‐time infected 2. It is plausible that the social and risk networks of recently infected persons might be relatively rich targets in which to find undiagnosed positives, both because these networks are likely to contain a disproportionate percent of newly‐infected people who have not yet had time to get tested, and thus diagnosed, and because they may be more likely to contain people with longer‐term undiagnosed HIV who may have been the sources of infection for the recently‐infected participants.

We therefore hypothesized: (1) that the networks of recently‐infected people are more likely to include members with undiagnosed HIV infection than are the networks of people with longer‐term infection; and (2) that recruiting people for testing in the networks of HIV‐infected people (whether recently‐infected participants or longer‐term infected) will yield a higher rate of undiagnosed positives than will standard outreach HIV testing methods.

Costs can affect the feasibility of interventions. We thus also compare the costs of locating an undiagnosed positive in TRIP with the costs of doing this, using standard testing methods.

## 2 Methods

We compared the yields of undiagnosed HIV positives from three projects. These are TRIP; the 2013 Integrated Biobehavioural Surveillance (IBBS) of people who inject drugs (PWID) in Odessa; and Outreach Testing sites in Odessa (funded by The Global Fund to fight AIDS, Tuberculosis and Malaria) whose data were captured in the Syrex program monitoring system from November 2013 through February 2016. We describe their methods below.

### 2.1 Setting

This research took place in Odessa, a large city in southern Ukraine, which is one of the areas where injection drug use and then an HIV epidemic began in the 1990s 21, 22 and then spread throughout the country. The HIV epidemic in Odessa, as in Ukraine generally, has been a concentrated one with PWID the key population with the most cases until 2008 15, 23, 24, 25, 26. Harm reduction programmes increased in size and effectiveness from 2004 and were showing signs of having leveled the epidemic off 27, though there are worries that recent social movements and the war in Eastern Ukraine may have reversed this.

### 2.2 TRIP methods

#### 2.2.1 TRIP eligibility criteria and arms

TRIP was a network intervention study with an intervention arm consisting of members of risk networks that were traced beginning with recently infected seeds, and a comparison arm consisting of risk network members of the longer‐term positive seeds. “Seeds” are people recruited as potentially recently‐infected who were interviewed and had specimens taken and whose risk network members were eligible for TRIP. “Risk networks” were operationalized as sex partners, people participants injected drugs with, people who were present while participants were having sex or using drugs, and people recruited from small‐size “venues” where participants went to inject drugs or locate sex partners. This extended definition of risk networks was used since it seemed likely to include others who might have been part of an infection chain that included the participant. (See Friedman *et al*. 2014 for a fuller explanation of this). Eligiblity criteria were age ≥18 years; ability to answer the questionnaire; and being qualified for one of the project arms.

The intervention arm consisted of recently‐infected seeds plus their risk network members. These seeds were index participants referred to TRIP by the Odessa City AIDS Center, the Odessa Regional Laboratory Center of the Ministry of Health of Ukraine, or the Way Home (a collaborating community organization) who were found to have recently been infected with HIV as follows: Recently‐infected seeds were generally defined as newly HIV‐diagnosed drug injectors (primarily) or others who had LAg OD_n_ ≤1.5 with viral load >1000 copies/ml or documented confirmed negative test within the prior six months. (Samples with LAg OD_n_ <0.4 were retested with Antigen/antibody tests as described below and if found positive were considered as recently‐infected participants unless they had viral load ≤1000 copies/ml).

The second (comparison) arm consisted of seeds with longer‐term HIV infections who were also used as seeds for network tracing (“longer‐term infected seeds”) plus their network members including any recently‐infected participants recruited as part of longer‐term infected networks and their networks. Longer‐term infected seeds” were recruited from the same referral sources as recently‐infected seeds. Longer‐term infected seeds were matched to recent seeds for age (±5 years), risk group and gender, and had LAg OD_n_ >1.5 without any evidence of seroconversion in the last six months.

For statistical analyses, only those recruited as network members (or from selected venues that participants attended) were analysed as being in the networks of recently‐infected participants, longer‐term infected seeds, or (for comparison with testing in IBBS and at Outreach Testing sites), either.

#### 2.2.2 TRIP assays

Blood samples were tested by New Vision Diagnostics Profitest Combo tests (Intec Products Inc., Haicang Xiamen, China) and confirmed by retesting with Profitest. Positive specimens underwent viral load assays using HIV‐1 Abbott RealTime™ (U.S. CORPORATE HEADQUARTERS, Abbott Laboratories, Abbott Park, IL, USA) and also were tested for recent infection with the Limiting Antigen Avidity (LAg) assay (Sedia™ Biosciences Corporation, Medical Technology, Portland, OR, USA) 28. LAg is based on antibody maturation and categorizes HIV infection as recent versus (vs.) longer‐standing. The standardized Optical Density (ODn) score of 1.5 was used as cut‐off for recency (130 days). Negative samples on LAg (OD_n_ <0.4) were retested with either HIV‐1/2 Ag+Ab‐Ultra MBA 0416/5 (MedBioAlliance, Kyiv, Ukraine) or with GenscreenUltra Ag/Ab, 6E0720 (BioRad, Marnes‐la‐Coquette, France) and if found positive were considered as recently‐infected participants unless they had viral load ≤1000 copies/ml.

#### 2.2.3 Dependent variable for TRIP

Participants were defined as undiagnosed positives if they tested HIV positive in the assays and if their interview indicated that the result of their most recent test was negative or unknown or if they had never been tested.

#### 2.2.4 Questionnaire

TRIP participants were interviewed with a questionnaire that included socio‐demographic characteristics, risk behaviours, and treatment history. Importantly, the questionnaire asked them to name their network members: people they injected or had sex with in the past six months; people who injected or had sex in their presence in the past six months; and people who injected, used drugs or had sex with people the participants had injected or had sex with. TRIP staff also asked them to indicate venues they usually visit to use drugs, to have sex, or to meet new sex partners.

#### 2.2.5 Network tracing in TRIP

The risk network members of recently‐infected and longer‐term infected seeds were recruited regardless of their infection status, as were the network connections of these network members. For those at network distance two from the seed (i.e. network members of network members), further network recruitment only occurred for those who were recently infected; for these, we continued recruitment of their network connections (to distance two) in similar fashion.

Network members who were recruited were tested for HIV; if they were positive, we carried out LAg tests and quantified plasma HIV‐RNA. Recently‐infected participants in networks were defined as people with documented testing history of recent infection (last negative–first positive test <6 months) irrespective of their LAg OD_n_ value; or with LAg OD_n_ ≤1.5 (and viral load >1000 copies/ml) if the testing history was unknown or their most recent test was both longer ago and negative. Longer‐term infected network members were those not classified as recently‐infected in seeds’ networks.

If a recently‐infected person was found in networks of seeds, the network members of that participant were recruited for two additional steps.

#### 2.2.6 Incentives and benefits of participation in TRIP

TRIP participants were given 50 hryvnia (approximately US$6 in 2013; US$2 in 2016) for baseline interviews and follow‐up interviews; 20 hryvnia (approximately US$2.50 in 2013; approximately $0.80 in 2016) for every named network member who brought in a referral coupon from the participant; and 10 hryvnia (approximately US$1.25 in 2013; approximately $0.40 in 2016) for every person recruited from a venue the participant named during the interview. The numbers of nominated network members and of venue members whom our staff recruited was not limited.

The project staff educated affected communities about recent/acute HIV infection, and about the importance of avoiding stigma. Participants were provided with standard counselling and were actively linked to care if appropriate.

### 2.3 IBBS methods

#### 2.3.1 IBBS overview

IBBS among PWID was a cross‐sectional respondent‐driven sampling (RDS) study in 29 cities of Ukraine in 2013, including Odessa. The sample size in Odessa city was 400 PWID. Trained and experienced interviewers conducted face‐to‐face interviews. The questionnaire was an adapted version of the IBBS questionnaire previously used among PWID in Ukraine (2007 to 2011) and contained questions about socio‐demographic characteristics, injection and sexual behaviour, previous HIV‐testing experience, etc. Experienced medical workers provided HIV rapid tests after the interview for all participants.

#### 2.3.2 IBBS‐dependent variable

In IBBS, participants were defined as undiagnosed positives if they tested HIV positive during the IBBS testing and if they self‐reported “negative” or “unknown” result on previous HIV tests or that they had never had a previous HIV test.

#### 2.3.3 IBBS eligibility and recruitment

Participants were enrolled in IBBS following preliminary screening based on the following criteria: at least 14 years old, had injected drugs within the last 30 days, currently resided in Odessa and non‐participation in any other surveys within the last six months. In addition, a medical worker checked veins for signs of punctures and only PWID with visible punctures were allowed to participate. Prior to enrollment into the study, both seeds and secondary respondents were provided with comprehensive information about the study and signed a consent form. Seeds were given three coupons to give to other PWID who could then take part in the study and receive compensation, as described below.

IBBS participants received compensation for their participation with 30 hryvnia (US$4 in 2013), plus 20 hryvnia (US$2.50) for the recruitment of each secondary participant according to RDS methodology.

#### 2.3.4 IBBS assays

All participants were tested for HIV by rapid test‐kit Immuno chromatographic assay to diagnose HIV Type 1 and Type 2 and subtype 0/CITO TEST HIV 1/2/0. Dry blood spot specimens were collected from all participants who had HIV‐positive rapid test results; these specimens were tested in Atlanta by the United States Centers for Disease Control and Prevention by two third generation HIV diagnostic ELISAs to confirm the presence of HIV antibodies (Abbott ARCHITECT HIV Ag/Ab Combo and Bio‐Rad Genscreen Ag/Ab HIV Ultra). Samples that tested reactive on both ELISAs were confirmed for HIV seropositivity using Western blot (Inno‐lia HIV‐1/2 Score, Innogenetics, Belgium).

#### 2.3.5 IBBS convergence diagnostics

RDS convergence was assessed. There were 13 waves. Diagnoses of the IBBS data were observed visually with convergence and bottleneck plots. Convergence plots for all variables of interest showed that estimates of all variables of interest appeared to be stable for the second half of the sample, with one exception: education reached convergence at the end of the sample 29.

The bottleneck plots for all variables appeared to converge on the point estimate. Additionally, recruitment and population homophily for all variables of interest fell in a range of estimates of 0.93 to 1.35 and 0.99 to 1.18 respectively.

### 2.4 Outreach Testing methods

#### 2.4.1 Outreach Testing overview and dependent variable

The Outreach Testing for HIV took place at community‐based harm reduction sites and at mobile vans in community settings. Testing was offered to all PWID reached by the NGOs conducting the outreach who self‐reported that they had never been tested for HIV or that they had been tested more than six months before and gotten a negative test. Thus, we defined all Outreach Testing clients who tested positive as being an undiagnosed positive. In terms of comparisons with the other projects, this exclusion of already‐diagnosed HIV positives is a conservative bias in that it may increase the proportion of Outreach Testing participants who are undiagnosed positives.

During the period November 2013 to February 2016 for which data were analysed, Outreach Testing programmes were modified in ways that affected their costs. Before 2015, they were conducted by doctors and nurses working for community based organizations. Thereafter, Outreach Testing was conducted by trained outreach workers who offered syringes and condoms to clients as well as HIV testing, and the numbers of clients being tested increased.

#### 2.4.2 Outreach Testing recruitment

Potential clients came to the sites where HIV testing was conducted. In addition, if outreach social workers learned of new locations where potential clients gather, they would go to those venues to recruit people to become clients and to be tested. Data collected about clients included their age, sex, years of injection drug use and risk group; but many variables that TRIP and IBBS include are not available for Outreach Testing.

#### 2.4.3 Outreach Testing services

Outreach Testing clients did not receive any incentives for their testing within the programme. They did, however, receive harm reduction services, and those with HIV‐positive results were referred to case management and HIV treatment services.

#### 2.4.4 Outreach Testing assays

##### Assays

Outreach Testing used the following rapid test‐kits for HIV testing: New Vision Diagnostics “Profitest” Rapid Anti‐HIV (New Vision Solutions Ltd, Dhaka, Bangladesh), CITO TEST HIV 1/2/0 (PHARMAS CO Ltd, Vyshgorod, Kyiv region, Ukraine) and SD BIOLINE HIV 3.0 (Standard Diagnostics, Inc., Yongin‐si, Gyeonggi‐do, Korea).

##### Cost comparison analysis

Cost comparison analysis used a simplified ingredients‐based approach 30: Data on staffing costs, recruitment costs, and costs of processing assays, all of which were available through Alliance for Public Health administrative data, were compared across TRIP, Outreach Testing (before 2015 and thereafter) and IBBS to calculate the costs for each project. These totals were then divided by the numbers of undiagnosed positives detected by each project to calculate their cost per new diagnosis located. Both IBBS and TRIP involve substantial research components whose costs were excluded for these comparisons since they are not part of the intervention.

##### Analyses

Since TRIP recruited both PWID and non‐PWID, comparisons between TRIP arms and comparisons of TRIP with IBBS and Outreach Testing included analyses stratified by injection drug use. Cross‐tabulations and frequencies were calculated using SPSS version 21. Odds ratio (OR) and their confidence intervals were calculated from cross‐tabulations using formulas from Daniel [31, p. 639]. Daniel defines the estimated OR for a cross‐tabulation table OR = (a/b)/(c/d), and constructs a confidence interval as =100(1−α)%CI=OR^(1±(zα(χ2)), where z_α_ is the two‐sided z value corresponding to the chosen confidence coefficient and χ^2^ is computed as = (n(ad − bc)^2^/(a + c)(b + d)(a + b)(c + d)), with a, b, c, d being the cells in the cross‐tabulation and n the number of cases. Since none of the data are based on probability samples, the confidence intervals should be viewed as heuristic estimates. Questions of how well TRIP worked for subsets such as women or PWID are primarily addressed through stratified analyses. In addition, to check whether there is additional confounding that might affect the magnitude of these OR, multiple logistic regression was used to compute adjusted OR as a secondary analysis.

##### Human subjects

TRIP participants gave informed consent under protocols approved by the IRBs of the National Development and Research Institutes and the Medical Ethics Committee at Gromashevsky Institute of Epidemiology and Infectious Diseases. IBBS participants gave informed consent under protocols approved by Sociology Association of Ukraine and Medical Ethics Committee at Gromashevskii Institute of Epidemiology and Infectious Diseases. In addition, the incidence component of IBBS was approved by the Medical Ethics Committee at Gromashevskii Institute of Epidemiology and Infectious Diseases. Outreach Testing data are program data about the participation of clients seeking health‐related services and did not require an informed consent form.

## 3 Results

The TRIP sample contained both PWID (45.1%) and non‐PWID. IBBS was, by design, composed only of injectors, and Outreach Testing also included only injectors. Nonetheless, the TRIP and IBBS samples had similar distributions by sex; all three had a median age of approximately 35; TRIP and IBBS had similar proportions of high school completers, and, for participants who inject, TRIP and IBBS had about 15 as the median years of injection while Outreach Testing injectors had a median ten years of experience (Table 1). The TRIP sample had higher rates of homeless and unemployed than did IBBS, and had a higher rate (among PWID) who were in drug or alcohol treatment. Within the TRIP sample, differences between the undiagnosed seropositives and previously‐diagnosed HIV‐infected participants were small except that the undiagnosed were less likely to be on drug or alcohol treatment (data not shown).

**Table 1:** Characteristics of participants in TRIP networks (combining the networks of recently infected participants and of longer‐term infected), IBBS, and Outreach Testing in Odessa

	TRIP networks total	TRIP networks PWID only	IBBS (% weighted for RDS sampling)	Outreach Testing
Total	1252	551	400	13,936^a^
Males	993 (79.3%)	471 (85.5%)	328 (82.0%)	9669 (69.4%)
Median age in years (IQR)	34 (27 to 41)	35 (29 to 41)	35 (29 to 42)	35 (30 to 39)
Education—at least high school (11 years) completed	980 (78.3%)	434 (78.8%)	315 (78.8%)	Not available
Homeless	168 (13.4%)	54 (9.8%)	1 (0.3%)	Not available
PWID^c^ (injecting over the last six months)	551 (44.0%)	551 (100%)	400^c^ (100%)	Not available
Median duration of injection in years (IQR)	Not applicable, see next column	15 (7 to 21)	16 (10 to 22)	10 (7 to 14)
On drug/alcohol treatment at enrollment	102 (8.1%)	54 (9.8%)	9 (2.3%)	Not available
Unemployed/unable to work	496 (39.6%)	256 (46.5%)	89 (22.3%)	Not available
Sex workers	4 (0.3%)	2 (0.4%)	0%	Not available
Male sex workers (% of males)	1 (0.1%)	1 (0.2%)	0%	Not available
Female sex workers (% of females)	3 (1.2%)	1 (1.3%)	0%	Not available
HIV prevalence rate^b^	329 (26.3%)	186 (33.8%)	108 (27.0%)	331 (2.4%)
Percent who are newly diagnosed HIV positive	183 (14.6%)	103 (18.7%)	20 (5.0%)	331 (2.4%)

RDS, respondent‐driven sampling; TRIP, Transmission Reduction Intervention Project; IBBS, Integrated Biobehavioural Surveillance; PWID, people who inject drugs.

a N tested for HIV during the period in Outreach Testing, out of 23,204 PWID who were covered by harm reduction services.

b Four PWID had indeterminate HIV test results.

c IBBS and Outreach Testing participants were all PWID.

Data on proportions of undiagnosed positives in each project appear in Table 2. OR comparing the projects in terms of the percent of participants tested for HIV who were previously undiagnosed positives appear in Table 3. As can be seen, TRIP (and both its recently‐infected participants’ networks and longer‐term positives’ networks) had higher yields of undiagnosed positives than did either the RDS surveillance in IBBS or Outreach Testing. These findings remained significant with high OR even when only the PWID in the TRIP samples were compared with Outreach Testing and IBBS, and when the OR were restricted to the men in the sample.

**Table 2:** Numbers and percentages who are newly diagnosed as HIV positive in 1. TRIP network‐participants by network type; 2. in IBBS; and 3. in Outreach Testing samples^a^^,^ b

	N tested for HIV	HIV−	HIV+	Newly diagnosed	% Newly diagnosed among those tested
1. Total TRIP networks (adding recently infected participants’ networks together with LT+s’ networks**)**	1252	923	329	183	14.6%
Women only	259	168	91	49	18.9%
Men only	993	755	238	134	13.5%
TRIP networks of recent seeds (all)	735	551	184	120	16.3%
PWID only	303	201	102	63	20.8%
Non‐injectors only	432	350	82	57	13.2%
Homeless only	110	88	22	17	15.5%
Non‐homeless only	625	463	162	103	16.5%
Women only	156	96	60	34	21.8%
Men only	579	455	124	86	14.9%
PWID women	50	20	30	17	34.0%
PWID men	253	181	72	46	18.2%
TRIP networks of longer‐term positive seeds	517	372	145	63	12.2%
PWID only	248	164	84	40	16.1%
Non‐injectors only	269	208	61	23	8.6%
Homeless only	58	45	13	7	12.1%
Non‐homeless only	459	327	132	56	12.2%
Women only	103	72	31	15	14.6%
Men only	414	300	114	48	11.6%
PWID women	30	18	12	6	20.0%
PWID men	218	146	72	34	15.6%
2. IBBS (unweighted) (All are PWID)	400	292	108	20	5.0%
Women only	72	48	24	5	6.9%
Men only	328	244	84	15	4.6%
3. Outreach Testing November 2013 to March 2016 (All are PWID)	13,932^a^	13,601	331	331	2.4%
Women only	4266	4179	87	87	2.0%
Men only	9666	9422	244	244	2.5%

TRIP, Transmission Reduction Intervention Project; IBBS, Integrated Biobehavioural Surveillance; PWID, people who inject drugs.

a All participants in the Outreach Testing sample were previously negative by self‐report.

b Four PWID had indeterminate test results and were excluded from these analyses.

**Table 3:** Odds ratios and 95% confidence intervals^a^ for comparing proportions newly diagnosed as HIV+ in TRIP (columns) with IBBS and Outreach Testing (rows)^a^^,^ b

Total	TRIP	TRIP networks of recently‐infected participants	TRIP networks of long‐term positives
IBBS	3.25 (2.07, 5.12)	3.71 (2.33, 5.89)	2.64 (1.59, 4.37)
Outreach Testing	7.03 (5.95, 8.31)	8.02 (6.62, 9.71)	5.70 (4.24, 7.35)
PWID only			
IBBS	4.37 (2.74, 6.96)	4.99 (3.06, 8.14)	3.65 (2.14, 6.24)
Outreach Testing	9.45 (7.74, 11.5)	10.8 (8.48, 13.7)	7.90 (5.85, 10.7)
Women only			
IBBS	3.13 (1.25, 7.83)	3.73 (1.47, 9.49)	2.28 (0.81, 6.45)
Outreach Testing	11.2 (8.25, 52)	13.4 (9.51, 18.9)	8.19 (4.99, 13.4)
Men only			
IBBS	3.26 (1.93, 5.49)	3.64 (2.13, 6.22)	2.74 (1.53, 4.88)
Outreach Testing	6.02 (4.94, 7.34)	6.74 (5.36, 8.47)	5.06 (3.77, 6.80)

TRIP, Transmission Reduction Intervention Project; IBBS, Integrated Biobehavioural Surveillance; PWID, people who inject drugs.

a The N's from which this table was derived appear in Table 2.

b Since the samples are not probability samples, the confidence intervals are heuristic estimates.

To test whether personal characteristics of participants might affect the OR for comparisons between TRIP and IBBS, logistic regression analyses controlling for age, gender, injection drug use, how many years they had been injecting drugs, homelessness, unemployment, sex work, and being in drug treatment were conducted. All adjusted OR were similar in magnitude to the unadjusted OR, and all statistically significant results remained significant. We did not conduct these analyses for comparisons with Outreach Testing participants due to the very limited available data on their personal characteristics.

Within TRIP (Table 4), the recently‐infected participants’ networks had a higher yield of undiagnosed positives than did the longer‐term positives’ networks. Within subsets of participants, recently‐infected participants’ networks contained higher rates of undiagnosed positives only among a. non‐injectors and b. participants with homes. Among PWID, homeless, female and male subsamples, the OR were all >1.40, but their confidence intervals overlapped unity.

**Table 4:** Odds ratios and 95% confidence intervals for showing the extent to which the TRIP networks of recently‐infected participants located more undiagnosed HIV positives than the TRIP networks of longer‐term positive seeds

	Odds ratios (95% confidence interval)
Total	1.41 (1.01, 1.95)
PWID only	1.36 (0.88, 2.11)
Non‐PWID only	1.63 (0.98, 2.70)
Homeless only	1.33 (0.52, 3.42)
Not homeless	1.42 (1.00, 2.01)
Women	1.63 (0.84, 3.17)
Men	1.33 (0.91, 1.94)

TRIP, Transmission Reduction Intervention Project; PWID, people who inject drugs.

When multiple logistic regression was conducted to determine if the OR in Table 4 changed when controlling for personal characteristics, two changes were noted. The adjusted OR within the non‐injector subsample became statistically significant, and that within the homeless subsample increased to 2.86 while remaining marginally non‐significant (*p* = 0.068). All other comparisons remained similar to those in Table 4.

Table 5 presents the cost comparison calculations. TRIP located undiagnosed positives at much lower cost ($250) than either of the other projects (IBBS $387; Outreach Testing $941 in the early period; $653 thereafter).

**Table 5:** Cost comparison^a^

	Items	Comments	Cost, US$	Quantity	Total cost, $
TRIP November 2013 to March 2016					
Staff costs storefront	Interviewer	53 interviews per month; 33 hours per week per person; 2 persons	10.55	1452	15320.05
	Social worker per month	25 h per week	136.77	28	3829.62
Medical staff	Nurse per month	4 per day	117.23	28	3282.53
Recruitment costs	Interview		1.95	1452	2837.05
	Contact		0.78	1452	1134.82
	Place		0.39		
Test procurement	Rapid test	For detection	1.00	1452	1452.00
	Rapid test	In Lab for HIV+	1.00	356	356.00
	LAg	Per test	10.89	356	3878.30
	Viral load	Per test	22.26	356	7926.38
Lab labour	LAg	Per test conducted	3.13	356	1112.93
	Viral load	Per test conducted	5.86	356	2086.75
Total cost					43,216.43
Number of people tested				**1452**	
Number of HIV+				**356**	
Number of undiagnosed HIV positives detected				**173**	
Cost per undiagnosed positive detected					249.81
IBBS (38 days of actual data collection)					
Site staff	Interviewer		5.00	400	2000.00
	Coupon manager	Per month	500.00	2	1000.00
Medical staff	Nurse	Per test	4.38	400	1750.00
Recruitment cost	Interview		4.00	400	1600.00
	Recruiting		2.50	400	1000.00
Test procurement	Rapid test	For detection	1.00	400	400.00
Total cost per period					7750.00
Number of people tested				**400**	
Number of HIV+				**108**	
Number of undiagnosed HIV positives detected				**20**	
Cost per undiagnosed positive detected					387.50
HIV Outreach Testing November 2013 to December 2014^b^					
Site staff	Outreach/social worker		300.00	406	121,800.00
Medical staff	Doctor	Per month	178.30	112	19,969.60
Test procurement	Rapid test	For detection	1.00	5956	5956.00
Total cost per period					147,725.60
Number of people tested				**5956**	
Number of HIV+				**157**	
Number of undiagnosed HIV positives detected				**157**	
Cost per undiagnosed positive detected					940.93
HIV Outreach Testing January 2015 to February 2016^b^					
Site staff	Outreach/social worker		234.47	406	95,193.43
Test procurement	Rapid test	For detection	1.00	9960	9960.00
Total cost per period					105,153.43
Number of people tested				**9960**	
Number of HIV+				**161**	
Number of undiagnosed HIV positives detected				**161**	
Cost per undiagnosed positive detected					653.13

TRIP, Transmission Reduction Intervention Project; IBBS, Integrated Biobehavioural Surveillance. Bold values indicate critical parts of the table.

a Assumption: The analysis used average exchange rates of Ukrainian hryvnia to the dollar for the period during which each service was provided.

b As noted in the text, the organization of Outreach Testing changed at the end of December, 2014.

## 4 Discussion

Locating undiagnosed HIV positives was a public health priority even before Frieden et al. emphasized the importance of this in 2005 32. Its importance was also highlighted by findings that starting antiretroviral treatment (ART) early benefits HIV‐infected people and reduces sexual HIV transmission 33, 34, 35. Locating undiagnosed positives is also critical for assuring that 90% of those who are infected know they are, which is the first step in the 90‐90‐90 strategy that underpins most HIV research and intervention in recent years 1.

Recruiting the risk networks of infected people in TRIP led to locating a higher rate of undiagnosed positives than did either RDS recruitment (IBBS) or Outreach Testing. This may in part be due to TRIP's success in recruiting more (self‐reportedly) homeless and unemployed participants (partly as a result of venue targeting, possibly also due to different reimbursement rates for participants’ time) than IBBS. (Data on homelessness and unemployment are not available for Outreach Testing). Within TRIP, a higher proportion of undiagnosed positives were located in networks of recently‐infected seeds than in networks of seeds with longer‐term infection.

The superiority of TRIP as a way to recruit undiagnosed positives probably stems from its design. TRIP recruits people who either engaged in risk behaviour with infected people or who are socially, sexually, or injection‐linked with recently‐ or longer‐term infected seeds. This contrasts with RDS (which IBBS used), which attempts to recruit a probability sample of PWID by having participants recruit other PWID (though not necessarily people they inject with). Thus, TRIP zeroes in on the social networks most likely to be infected (and in one arm, on those likely to be recently infected and undiagnosed) whereas IBBS attempts to use “weak ties” to recruit PWID who are socially and geographically distant from the seeds. TRIP also contrasts with Outreach Testing which recruits those who come in off the streets.

Epidemiologically, these findings support our hypothesis that the risk networks of the infected—and particularly those of the recently‐infected—contain a higher proportion of undiagnosed positives than do the samples reached either in RDS surveillance of the kind done by IBBS programs or in Outreach Surveillance of PWID in their neighbourhoods. It is possible that the superiority of TRIP methods may depend on the phase of the local epidemic and/or on the history of testing in the community and/or access to testing for different population sub‐groups.

These findings are subject to limitations. Samples that were HIV negative by the rapid test were not sent for confirmatory testing. This could have resulted in underestimation of numbers of infected people and thus perhaps in underestimation of the number of undiagnosed positives. Such an underestimate is not likely to be large, however, since the rapid test has sensitivity and specificity over 99% 36. Another limitation is that TRIP, IBBS and Outreach Testing all rely on self‐reports to determine which participants who test positive have not been previously diagnosed. Furthermore, the questions used to do this in the three studies were not identical. Similarly, TRIP defined injectors as those who had injected drugs in the previous six months; IBBS in the previous 30 days. IBBS gave out only three coupons, and TRIP gave coupons depending on size of self‐reported risk networks. Error could also be introduced into comparisons between the arms of TRIP if staff put extra effort into recruiting recently‐infected participants’ network members, although the directionality of such an effect on the proportion of people tested who were undiagnosed positives is uncertain. Outreach Testing rates for the percent of clients who were undiagnosed positives may have been inflated by excluding those who already knew they were infected; outreach programmes that do not exclude such clients might have slightly lower yield rates. Another limitation is that the cost comparison was limited by difficulties in estimating non‐intervention research expenses and excluding management, facility‐related and administrative costs, as well as by the change in the exchange rate of the hryvnia from eight to the dollar to over 25 to the dollar during the study. Since salaries and most other expenses were not adjusted, this means that (if measured in dollar terms), TRIP had even more of a cost advantage over IBBS than we estimated. Another potential limitation is that both TRIP and IBBS depend on monetary incentives to recruit participants; additional research is needed to determine if they work without such incentives and about the best levels of incentives to use. Finally, the overall effectiveness of TRIP methods (as well as of other techniques) in locating undiagnosed positives may depend on levels of stigma against HIV‐infected people and on the scale and barriers of access to other testing sites.

Implementing TRIP methods on a public health scale faces a challenge: It is not easy to locate enough original recently‐infected seeds (except perhaps in a very rapid outbreak with very high seroincidence rates). We suggest that TRIP‐style interventions be implemented as supplements to existing HIV testing programmes in which these programmes would use data on previous negative tests for those who test positive and also use Limiting Antigen Avidity Assay or a similar test for recent infection for people who they think might have been recently infected. A rapid test might make this process easier because there would be no need to ask them to return to the test site to be interviewed. Such probable seeds could then be brought to the attention of an area‐wide TRIP team to conduct the enhanced risk network tracing intervention. Such teams might work in conjunction with standard contact tracing teams 37, 38.

## 5 Conclusions

TRIP recruited members of the risk networks of people recently‐infected with HIV and located a higher rate of undiagnosed positives than other programmes in Odessa. This finding has important implications for public health practice. Together with conceptually‐similar findings that the TRIP intervention recruits high rates of recently‐infected PWID in Athens 2 and a study in San Diego 3 that found that contact tracing of sex partners of people with acute or early HIV infection in San Diego was effective in finding undiagnosed HIV positives, our results strongly suggest that network recruiting techniques similar to those used in TRIP should become part of standard case finding and treatment as prevention practice. Research is needed to determine if these findings hold true in other social and epidemiologic contexts, as well as to find ways to improve the field performance and cost effectiveness of such network interventions.

## Competing interests

None to declare.

## Authors’ contributions

PS Helped write the funding proposal; oversaw and contributed to all TRIP field operations; helped conceptualize this paper; conducted the analyses of costs; helped write the paper; and approved the final version. LW Contributed to TRIP field operations; helped conceptualize this paper; conducted most of the statistical analyses; helped resolve complex data issues; helped write the paper; and approved the final version. AK Managed day to day TRIP field operations during much of the project; organized and maintained complex data files; helped conceptualize this paper; helped resolve complex data issues; contributed to the analyses; helped write the paper; and approved the final version. YS Conducted analyses of the IBBS and Outreach Testing data; helped write the paper; and approved the final version. GKN Helped conceptualize this paper; helped design the analysis plan; helped write the paper; and approved the final version. BS Helped conceptualize this paper; helped write the paper; and approved the final version. EM Helped conceptualize this paper; helped write the paper; and approved the final version. JS Helped write the proposal; helped conceptualize this paper; helped write the paper; and approved the final version. TIV helped write the funding proposal; managed day to day field operations during much of the project start‐up; set up and organized complex data files; helped write the paper; and approved the final version. SRF Overall project principal investigator; oversaw all field operations in some detail; wrote funding proposal with help from PS, JS and TIV; led in conceptualizing and directing this paper; conducted some analyses; helped write the paper; and approved the final version.

## Funding

This intervention research was supported by the United States (US) National Institute on Drug Abuse (NIDA) grants DP1 DA034989 and P30DA011041. BS was also supported by NIH Research Training grant T32AI7384‐26. JS was also supported by grants R01 DA033875 and R21 AI118998 from the National Institutes on Drug Abuse and the National Institute of Allergy and Infectious Diseases, respectively. TIV was supported by the Clarendon Fund of the University of Oxford. The funding sources had no role in the design and conduct of the study; collection, management, analysis, and interpretation of the data; preparation, review, or approval of the manuscript; and decision to submit the manuscript for publication.
